# Bioinspired pH-sensitive liposomes for quercetin delivery to synergize with 5- FU in gastric cancer therapy

**DOI:** 10.1016/j.ijpx.2025.100437

**Published:** 2025-10-31

**Authors:** Qinghua Lan, Miao Wang, Yanyan Zhu, Xiayan Zhang, Ruolei Ye, Zhengbo Wu, HaiCi Lan, Songmei Luo, Yanyan Xu

**Affiliations:** a Department of Pharmacy, The Fifth Affiliated Hospital of Wenzhou Medical University, Lishui 323000, China; b Key Laboratory of Imaging Diagnosis and Minimally Invasive Intervention Research, The Fifth Affiliated Hospital of Wenzhou Medical University, Lishui 323000, China; c School of Pharmacy, Hangzhou Medical College, Hangzhou 310053, China; d College of Food and Health, Zhejiang Agricultural and Forestry University, Hangzhou 313000, China; e Research Institute of Pharmaceutical Sciences, College of Pharmacy, Chonnam National University, Gwangju 61186, Republic of Korea

**Keywords:** Gastric cancer, pH-sensitive liposomes, Quercetin, 5-Fluorouracil, Synergistic treatment

## Abstract

Gastric cancer is a major cause of cancer-related mortality on a global scale. Although 5-fluorouracil (5-FU) is a cornerstone chemotherapeutic for digestive tract malignancies, its efficacy is limited by dose-dependent toxicity and acquired resistance. Quercetin (QUC), a natural flavonoid, can sensitize tumor cells to 5-FU by modulating cell cycle-regulatory proteins. However, its limited water solubility and low bioavailability present significant limitations on its potential therapeutic application. In this study, we developed bioinspired pH-sensitive liposomes (NK-Lip@Q) functionalized for active targeting and acid-triggered drug release to enhance QUC delivery and synergistic anticancer activity with 5-FU. NK-Lip@Q exhibited a mean particle size of 206.36 ± 1.81 nm, an encapsulation efficiency of 60.69 ± 1.32 %, and a pH-dependent release profile with 72.75 ± 0.69 % cumulative release at pH 5.4. Cellular studies demonstrated efficient uptake by N87 cells, marked apoptosis induction (apoptosis ratio: 69.60 ± 8.71 %), and enhanced cytotoxicity in combination with 5-FU (Chou-Talalay combination index, CI = 0.68). In vivo, NK-Lip@Q could precisely accumulate in the target area, when co-administered with 5-FU, achieved significant tumor inhibition (tumor inhibition rate: 92.26 %) without obvious systemic toxicity. QUC complemented the anticancer action of 5-FU by regulating cell cycle-related genes, promoting apoptosis, and suppressing proliferation. In conclusion, this study demonstrates that NK-Lip@Q as a promising nanocarrier system that enhances the therapeutic performance of 5-FU by improving its synergistic antitumor efficacy in gastric cancer.

## Highlights

• Bioinspired NK cell membrane-fused liposomes enable targeted quercetin delivery.
• NK-Lip@Q exhibited pH-responsive release with about 72.75 % QUC cumulative release at pH 5.4.
• Combined NK-Lip@Q and 5-FU treatment exhibited a synergistic effect, with a Chou-Talalay combination index of 0.68.
• In vivo, NK-Lip@Q + 5-FU achieved 92.26 % tumor inhibition without obvious systemic toxicity.
• The NK-Lip@Q + 5-FU system offers a synergistic and biocompatible approach for gastric cancer.

## Graphical abstract

**Figure:**
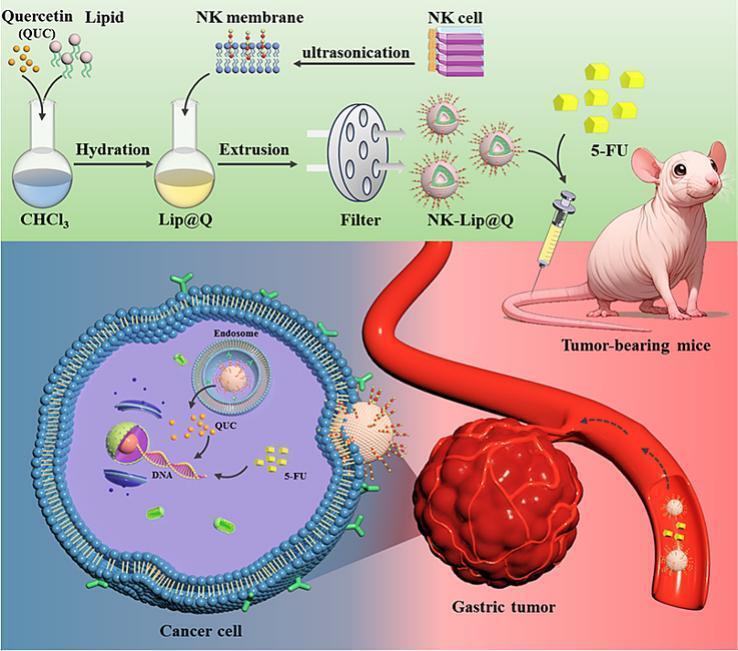

## 1 Introduction

Gastric carcinoma is a prevalent and lethal type of cancer that is widely distributed around the world. It is the fourth most common cancer in terms of incidence and the second in cancer-related mortality (Cai et al., 2024; Liao et al., 2023). Among the available chemotherapeutic regimens, 5-fluorouracil (5-FU)-based combination therapy is widely employed for advanced gastric cancer owing to its broad-spectrum antitumor activity (Liu et al., 2024; Peng et al., 2024). However, due to the influence of cancer stem cells, recurrence, metastasis and angiogenesis, the development of 5-FU resistance has become increasingly prominent, severely limiting its clinical efficacy (Yuan et al., 2023). Therefore, the development of innovative therapeutic strategies that can prolong survival while mitigating 5-FU resistance is a matter of urgent necessity.

Quercetin (3,3′,4′,5,7-pentahydroxyflavone, QUC), a member of the flavonoid family, has been extensively investigated for its anti-tumor potential in digestive system malignancies, including gastric, esophageal, colorectal, and pancreatic cancer (Asgharian et al., 2021; Neamtu et al., 2022; Wang et al., 2022; Zúñiga-Hernández et al., 2024). A recent study demonstrated that the combined administration of QUC and 5-FU markedly suppresses interleukin-8 secretion in colorectal cancer cells. By downregulating the expression level of this pro-inflammatory chemokine, QUC can enhance 5-FU-induced apoptosis in tumor cell (Abosalha et al., 2024). Therefore, QUC may act as a chemoadjuvant to 5-FU, synergistically amplifying its antitumor efficacy and offering a promising combinatorial strategy for gastrointestinal malignancies. In addition to this synergistic effect, Tavakoli Pirzaman et al. (2023) found that moderate to high doses of QUC can significantly alter the pharmacokinetics of 5-FU, promoting its in vivo accumulation. This finding suggests that when co-administration of QUC may allow dose reduction of 5-FU, thereby improving patients' tolerance to cytotoxic therapy. Overall, quercetin exhibits minimal intrinsic toxicity and does not exacerbate the side effects of other anticancer drugs, making it an attractive candidate for combination chemotherapy.

Nevertheless, the poor aqueous solubility and extensive first-pass metabolism of QUC greatly limit its bioavailability, making it difficult to achieve therapeutically effective concentrations at the lesion target sites (Mirza et al., 2023). To overcome this limitation, we designed a biomimetic liposome delivery system (NK-Lip) tailored to the characteristics of the gastric tumor microenvironment for the targeted delivery of QUC. As an advanced nanocarrier, liposomes exhibit biomembrane-like properties and efficient drug transport capability (Fulton and Najahi-Missaoui, 2023; Pande, 2023). They not only improve the solubility of poorly water-soluble drug but also facilitate their extravasation through the leaky vasculature of tumor neovessels, enhancing drug accumulation at the lesion site. However, conventional liposomes are susceptible to recognition and clearance by the reticuloendothelial system during systemic circulation, thereby compromising their therapeutic efficacy (Escudé Martinez De Castilla et al., 2025; Papp et al., 2025).

Natural killer (NK) cells are considered to be pivotal elements of the innate immune system (Nabekura, 2025). These cells play crucial roles in antitumor immunity, antiviral defense and immune regulation, and may also participate in hypersensitivity and autoimmune reactions under certain conditions (Peris Sempere et al., 2024). By modifying liposomes with NK cell membrane, the resulting biomimetic liposomes inherit the membrane proteins of NK cells, which help them evade immune surveillance, prolong systemic circulation, and enhance tumor targeting capability.

**Scheme 1:**
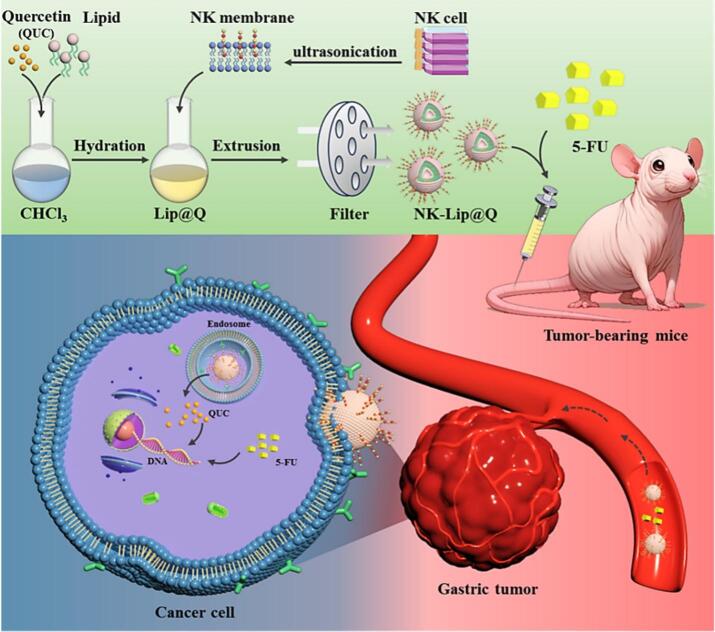
Schematic illustration of the preparation of NK-Lip@Q and its combinational therapeutic mechanism with 5-FU against gastric cancer. QUC was first encapsulated into conventional liposomes (Lip@Q) via the thin-film dispersion method. Subsequently, NK cell membranes were extracted and fused with the outer bilayer of Lip@Q to construct a biomimetic liposomes (NK-Lip@Q). Following intravenous administration, NK-Lip@Q was expected to actively target gastric tumor site and release QUC. Through this targeted delivery, NK-Lip@Q synergizes with 5-FU to enhance antitumor efficacy and reduce systemic toxicity.

In this study, we encapsulated QUC into NK cell membrane–modified liposomes (NK-Lip@Q) to achieve pH-responsive drug release within the acidic tumor microenvironment (Scheme 1). We hypothesize that NK-Lip@Q can enhance tumor accumulation, sustain localized drug release, and synergize with 5-FU to achieve superior antitumor efficacy in gastric cancer therapy. This biomimetic delivery approach offers a promising strategy for overcoming chemoresistance and improving the therapeutic outcomes of gastric cancer.

## 2 Materials and methods

### 2.1 Materials

Quercetin, cholesterol and egg-yolk lecithin were purchased from Solarbio (Beijing, China). MTT, membrane/cytosol extraction and annexin-V kits were supplied by Beyotime (Shanghai, China). FITC, DAPI and the TUNEL apoptosis reagent were obtained from Sigma-Aldrich (St. Louis, MO, USA) and Yeasen (Shanghai), respectively. Primary antibodies of anti-CCNB1, Anti-CDK1, Anti-CCNA2, and Anti-GAPDH were purchased from Proteintech (Chicago, USA).

### 2.2 Cell culture

N87, NCI-N87-LUC and NK-92MI cells, as well as their respective media and supplements, were purchased from iCell Bioscience (Shanghai, China) and Huashu Biotechnology (Hangzhou, China). N87 and NCI-N87-LUC were maintained in RPMI-1640 plus 10 % FBS and 1 % penicillin-streptomycin at 37 °C, 5 % CO₂; NK-92MI was grown in MEM-α containing 12.5 % horse serum, 12.5 % FBS and 1 % antibiotics under the same conditions.

### 2.3 Network pharmacology analysis and experimental validation

#### 2.3.1 Identification of differentially expressed genes(DEGs)

Gastric-cancer expression profiles were downloaded from the Gene Expression Omnibus (GEO) (http://www.ncbi.nlm.nih.gov/geo/) under accession numbers GSE27342(Cui et al., 2011), GSE33335 (Cheng et al., 2013), GSE56807 (Wang et al., 2014) and GSE63089 (Zhang et al., 2015). All four series were run on the Affymetrix HuEx-1_0-st platform (GPL5175). Differential expression between tumor and adjacent normal samples was computed with Bioconductor workflows in R 4.4.0, applying thresholds of *P* < 0.05 and |log_2_ FC| > 1.

#### 2.3.2 Network pharmacology

To identify quercetin-associated proteins, we mined three public repositories: Traditional Chinese Medicine Systems Pharmacology Database and Analysis Platform (TCMSP), Integrative Pharmacology-based Research Platform of Traditional Chinese Medicine (TCMIP), and SwissTargetPrediction. Overlapping genes with gastric-cancer signatures were extracted and displayed as Venn plots generated in R. Intersection proteins were uploaded to STRING to construct a PPI network, whose topology was subsequently characterized with NetworkAnalyzer (Hu et al., 2022). Functional interpretation, including Gene Ontology (GO) and Kyoto Encyclopedia of Genes and Genomes (KEGG) pathway enrichment analyses together with network rendering, was completed with Cytoscape and additional R scripts.

#### 2.3.3 Western blotting assay

Western blotting followed a published procedure with minor modifications (Lan et al., 2024). Briefly, N87 cells treated with QUC (6.25, 12.5, and 25 μg/mL) were lysed in RIPA buffer plus protease inhibitors, centrifuged and collected the protein supernatant. Equal amounts of protein from each group were separated by gel electrophoresis and subsequently transferred to PVDF membranes. The membranes were cut according to molecular weight and incubated with anti-CCNB1 antibody (28603–1-AP, 1:4000), anti-CDK1 antibody (10762–1-AP, 1:2000), and anti-CCNA2 antibody (18201–1-AP, 1:25000) and anti-GAPDH antibody (10494–1-AP, 1:10000). Protein bands were visualized by chemiluminescence imaging technology.

### 2.4 Extraction and purity assessment of NK cell membranes (NKCM)

Two approaches were evaluated for NK cell membrane (NKCM) extraction. Method 1: Following the procedure described by Kang et al. (2017), NK cells were disrupted by ice-cold sonication, debris was removed at 800 ×g, 4 °C, 10 min, and mitochondria pelleted at 10000 ×g, 15 min, yielding NKCM1. Method 2: NKCM2 was extracted relied on membrane and cytosol protein extraction kit following the supplier's protocol.

The purity of NKCM samples was evaluated by agarose gel electrophoresis: samples mixed with GelGreen were run at 170 V for 15 min and imaged on Tanon-5200 system (Shanghai, China). A small amount of residual DNA was detected in NKCM2 prepared by Method 2, whereas NKCM1 showed no detectable DNA contamination. Therefore, Method 1 was selected for subsequent experiments due to its higher membrane purity and procedural simplicity.

### 2.5 Preparation of NK-Lip@Q

The NKCM-modified quercetin-loaded liposomes (NK-Lip@Q) were prepared based on previously reported protocols with minor modifications (Zhao et al., 2019). Briefly, quercetin, egg yolk lecithin, and cholesterol were dissolved in dichloromethane at predetermined molar ratios. To remove the organic solvent by reduced-pressure rotary evaporation, forming a lipid film. Subsequently, added 5 mL PBS to hydrate the lipid film. Finally, the QUC-loaded liposomes (Lip@Q) were obtained by gentle agitation followed by ultrasonic dispersion under controlled bath temperature and sonication time. Lip@Q were subsequently integrating with NKCM that obtained in Section 2.4. Sequential extrusion through 400 nm and 200 nm polycarbonate membranes generated NK-Lip@Q vesicles whose surfaces modified with the native NK cell membrane.

### 2.6 Evaluation of NK-Lip@Q

#### 2.6.1 Verification of NKCM components on NK-Lip@Q

The presence of NKCM proteins on NK-Lip@Q was verified by SDS-PAGE analysis. NKCM, Lip@Q, and NK-Lip@Q samples were were boiled for 10 min in loading buffer, resolved on 10 % gels at 90 V, stained 30 min with Coomassie Blue, destained overnight, and imaged on a gel imaging system.

#### 2.6.2 Encapsulation efficiency and drug loading capacity

QUC content in Lip@Q and NK-Lip@Q was quantified by UPLC-MS/MS to derive encapsulation efficiency (EE%) and drug loading capacity (LC%). 1 mg was disrupted in 2 mL methanol by sonication, centrifuged (14,000 rpm, 15 min), and the supernatant injected for analysis. UPLC conditions: ACQUITY UPLC BEH C18 column (100 mm × 2.1 mm, 1.7 μm); mobile phase: water with 0.1 % formic acid (A) and acetonitrile with 0.1 % formic acid (B); column temperature: 40 °C; flow rate: 0.3 mL/min; injection volume: 2 μL; gradient elution program: 0 min, 90 % A; 1.5 min, 90 % A;2.0 min, 10 % A; 3.0 min, 90 % A. MS conditions: electrospray ionization (ESI) in positive ion mode; capillary voltage 2.5 kV; cone voltage 36 V; ion source temperature 250 °C; solvent gas temperature 600 °C. Masslynx V4.1 software (Waters, USA) handled data acquisition and processing.

#### 2.6.3 Particle size (PS), zeta potential (ZP) and morphology

The particle size distribution and zeta potential of NKCM, Lip@Q, and NK-Lip@Q were measured by the dynamic light scattering detector (DLS, Litesizer™ 500, Austria). The morphology of Lip@Q and NK-Lip@Q was observed using a transmission electron microscope (TEM, Hitachi HT7800, Japan). Moreover, the UV–visible spectrophotometer (TU-1901, Beijing) was used to analyze the absorption spectrum (200–600 nm) of the liquid sample.

#### 2.6.4 Drug release assay

Dialysis was used to monitor QUC release from NK-Lip@Q. 5 mg of freeze-dried liposomes were suspended in 1 mL PBS, sealed in a pre-activated dialysis bag, and incubated at 37 °C in 50 mL of buffer with gentle shaking. At set times, 2 mL of external medium was removed and replaced with fresh buffer; QUC content was determined by the UPLC-MS/MS protocol outlined in Section 2.6.2.

#### 2.6.5 Hemolysis test

Hemocompatibility of NK-Lip@Q was evaluated by a hemolysis assay. Heparinized mouse blood was washed with PBS and centrifuged (1000 rpm, 5 min) to obtain erythrocytes, which were diluted to 2 % (*v*/v) in PBS. Aliquots were mixed with PBS (negative control), water (positive control), QUC, Lip@Q or NK-Lip@Q and incubated at 37 °C for 24 h. After centrifugation, 100 μL of supernatant was transferred to a 96-well plate and absorbance read at 540 nm on a multimode reader.

### 2.7 In vitro cellular studies

#### 2.7.1 Cellular uptake evaluation

For uptake imaging, FITC replaced QUC to generate Lip@F and NK-Lip@F. N87 cells grown overnight on coverslips were exposed to the fluorescent formulations for 24 h, washed, fixed with 4 % paraformaldehyde, counter-stained with DAPI, and examined under an EVOS M5000 inverted fluorescence microscope (Invitrogen, USA).

#### 2.7.2 MTT assay

Cytotoxicity was measured by MTT. N87 cells seeded overnight in 96-well plates were exposed for 24 h to QUC, NK-Lip@Q, 5-FU (50 μg/mL) and 5-FU + NK-Lip@Q, respectively. Medium was then exchanged for 5 mg/mL MTT solution; after 4 h the crystals were dissolved in 100 μL DMSO and absorbance at 570 nm was read to calculate viability.

#### 2.7.3 Apoptosis detection assay

Apoptosis was assessed by flow cytometry. Log-phase N87 cells seeded in 6-well plates were treated for 24 h with QUC (25 μg/mL), NK-Lip@Q, 5-FU (50 μg/mL) and 5-FU + NK-Lip@Q, respectively. Following trypsinization and collection (1000 rpm, 3 min), cells were incubated 30 min in the dark with Annexin V-FITC/PI and immediately analyzed on using a flow cytometer (BD FACSVia, China).

#### 2.7.4 Colony formation assay

Long-term proliferation proliferative and self-renewal ability of N87 cells was examined by colony formation. Cells in each group was treated for 24 h as described in Section 2.7.3, harvested and reseeded into fresh 6-well plates with 1 × 10^4^ cells/well. After 14 days, colonies were fixed (4 % paraformaldehyde, 15 min), stained (1 % crystal violet, 10 min), rinsed, dried and imaged.

### 2.8 In vivo animal studied

#### 2.8.1 Animals

BALB/c nude mice (male, 6–8 weeks) were purchased from Hangzhou Huashu Biotechnology Co., Ltd. They were housed in SPF setting (25 ± 2 °C, 50 ± 5 % RH, 12 h light–dark). Procedures were approved by the Wenzhou Medical University Animal Ethics Committee (wydw2025–0409). All animal experiments were performed in compliance with the Guide for the Care and Use of Laboratory Animals and the ARRIVE guidelines. G*Power 3.1 (one-way ANOVA, f = 0.65, α = 0.05, power = 0.80) indicated *n* = 7 per group; 35 mice were therefore randomized to five groups, with every step taken to minimise suffering and animal numbers. The value of the effect size (f = 0.65) was referenced from Yuan et al. (2006), who reported the tumor inhibition rate of quercetin liposomes.

#### 2.8.2 Gastric cancer model establishment

An orthotopic gastric cancer model was established in male BALB/c nude mice following a modified protocol described by Cox et al. (2025). Briefly, NCI-N87-LUC cells were first inoculated subcutaneously to generate viable tumor tissue, which was then cut into small fragments (∼1 mm^3^) and implanted into the gastric wall under sterile conditions. Tumor growth in situ was monitored weekly using bioluminescence imaging (IVIS Lumina III, PerkinElmer, USA). Bioluminescent signals detected at 1 and 2 weeks post-implantation confirmed successful establishment of the orthotopic gastric cancer model.

#### 2.8.3 Anti-tumor effect study

Orthotopic tumor-bearing mice were randomly allocated to the following groups: QUC (20 mg/kg), NK-Lip@Q, 5-FU (10 mg/kg), NK-Lip@Q + 5-FU and control (0.9 % saline, 1 mL/kg). All agents were given intravenously every 3 days for seven cycles. Tumor growth was tracked weekly by in vivo bioluminescence imaging of the gastric region; pretreatment signal served as the baseline to calculate real-time efficacy. At endpoint, stomachs were excised for volume measurement followed by histological analyses.

During the study, mice were checked daily for weight, intake and behavior. Humane endpoints were defined as ≥20 % weight loss, food intake <0.5 g/24 h, water intake <1 mL/24 h, persistent hunching, or inability to feed or drink autonomously. Animals meeting any criteria were immediately euthanized under deep isoflurane anesthesia (4 % for induction, 2 % for maintenance, O₂ 2 L/min), followed by cervical dislocation.

#### 2.8.4 Tumor-targeting effect evaluation

To assess the in vivo tumor-targeting capability of NK-Lip@Q, DIR fluorescent dye was used in place of QUC to prepare Lip@D and NK-Lip@D. These formulations (equivalent DIR dose: 2 mg/kg) were administered to gastric cancer-bearing mice via tail vein injection according to the experimental groups (*n* = 3 per group). Fluorescence signal intensity of major organs was captured and quantified 24 h after injection.

### 2.9 Statistical analysis

All quantitative data are expressed as mean ± standard deviation (SD). Statistical analyses were performed using GraphPad Prism 8.3 Software (GraphPad Software, San Diego, CA, USA). Differences between two groups were assessed using Student's *t*-test was used for comparisons between two groups, while comparisons among three or more groups were analyzed by one-way analysis of variance (ANOVA) followed by Tukey's post hoc test. *P* value <0.05 was taken significant (^⁎^*P* < 0.05, ^⁎⁎^*P* < 0.01, ^⁎⁎⁎^*P* < 0.001).

## 3 Results and discussion

### 3.1 Network pharmacology-based identification and functional verification of QUC targets in gastric cancer

**Fig. 1:**
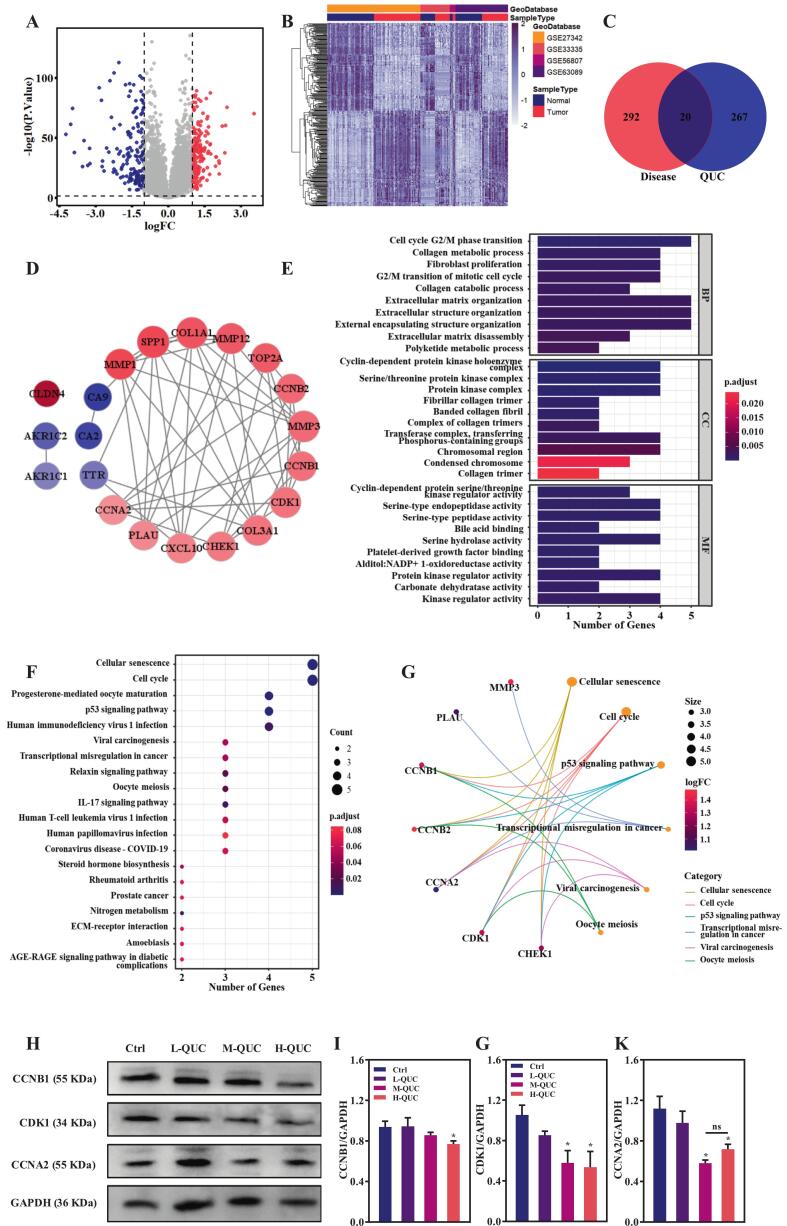
Identification of QUC's targets in gastric cancer via network pharmacology and experimental validation. (A) Volcano plot of DEGs: red indicated up-regulation, blue indicated down-regulation, and gray indicated normally expressed genes. (B) Heatmap of DEGs: purple indicated high expression levels, and grayish-white indicated low expression levels. (C) Venn diagram of the DEGs and the therapeutic target of QUC. (D) PPI network diagram of disease-drug intersection genes: the depth of color represents the level of differential expression. (E) GO enrichment analysis of disease-drug intersection genes. (F) KEGG enrichment analysis of disease-drug intersection genes. (G) Disease-drug intersection genes & pathway association network diagram. (H) Western blot for CCNB1, CDK1 and CCNA2 expression of N87 cells treated with L-QUC, M-QUC and H-QUC. Densitometry of CCNB1 (I), CDK1 (J), and CCNA2 (K) in western blots. (*n* = 3; * *P* < 0.05, ns = no significance, compared to Ctrl). (For interpretation of the references to color in this figure legend, the reader is referred to the web version of this article.)

A total of 310 samples were analyzed, comprising 155 gastric cancer tissues and 155 adjacent normal tissues. 312 DEGs were identified, including 163 upregulated and 149 downregulated genes (Fig. 1A&B). Overlaying QUC-specific targets with this DEG list produced 20 common genes (Fig. 1C). A PPI network constructed through the STRING database highlighted MMP1, SPP1 and COL1A1 as core targets with the highest degree values and relatively significant differential expression (Fig. 1D). GO enrichment and KEGG pathway analyses further revealed that these DEGs converge on cell cycle regulation, particularly in the G2/M phase transition, cyclin-dependent kinase (CDK) activity, and cellular senescence (Fig. 1E&F). These findings suggest that QUC may inhibit gastric cancer progression by disrupting cell cycle regulation. Consistent with this hypothesis, CCNB1, CDK1, and CCNA2 were identified as key regulators in the cell cycle pathway (Fig. 1G). Western blot analysis confirmed that high-dose QUC treatment markedly reduced the expression of these three cyclins in N87 cells (Fig. 1H–K), thereby impeding G2/M transition and slowing tumor proliferation. Notably, this result echoes earlier findings in liver and colon cancers, where QUC was shown to suppress tumor growth by downregulating the expression levels of cell cycle proteins and inducing cell cycle arrest (Sethi et al., 2023; Das et al., 2023). Collectively, this results indicate that QUC may exert a conserved antitumor mechanism across gastrointestinal malignancies by modulating cell cycle-related signaling networks.

### 3.2 Preparation and characterization of NK-Lip@Q

#### 3.2.1 Optimization of Lip@Q preparation parameters

**Fig. 2:**
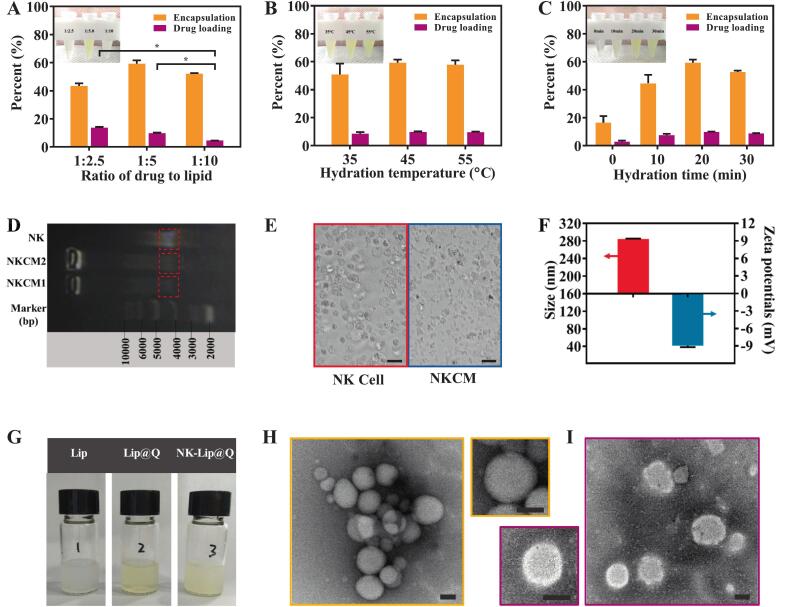
Formulation optimization, membrane characterization, and physicochemical properties of the liposomes. The encapsulation rate and drug loading capacity of Lip@Q were evaluated under varying ratios of QUC/lipids (A), hydration temperatures (B), and hydration times (C). (D) Agarose gel electrophoresis of NK cell and NK cell derived membrane reflecting purity of NKCM. (E) The images of NK cell and NKCM under the optical microscope. Scale bar: 25 μm. (F) Particles size and Zeta potentials of NKCM. (G) Optical images of Lip, Lip@Q and NK-Lip@Q. (H) TEM images of Lip@Q. (I) TEM images of NK-Lip@Q. (Scale bar: 100 μm; *n* = 3; * *P* < 0.05).

The preparation of Lip@Q represents the first step in constructing NK-Lip@Q. Among the key influencing factors, the QUC/lipid ratio, water bath temperature, and ultrasonic hydration time play crucial roles. To optimize the preparation process of Lip@Q, single-factor experiments were conducted using EE% and DL% of QUC as evaluation indices. Specifically, the effects of QUC/lipid ratio (1:2.5, 1:5, 1:10), water bath temperatures (35, 45, and 55 °C), and ultrasonic hydration times (0, 10, 20, and 30 min) were systematically investigated. Lip@Q prepared at a QUC/lipid ratio of 1:5 achieved the highest EE%, producing a pale-yellow solution (Fig. 2A). At a ratio of 1:2.5, drug loading was relatively higher but EE% was lower, and the solution appeared turbid yellowish-white after centrifugation. At 1:10, the liposome solution was highly unstable and exhibited phase separation within 1 h at room temperature. When the water bath temperature of 45 °C, EE value was the highest (Fig. 2B). Furthermore, an ultrasonic hydration time of 20 min gave maximal EE% (Fig. 2C). Compared with non-ultrasonic samples, ultrasonic hydration significantly enhanced EE (*P* < 0.001). This observation is consistent with the previous report demonstrating that ultrasonic treatment, via cavitation and shear effects, can increase liposome entrapment of QUC (Qu et al., 2025). Thus, we selected a QUC/lipid ratio of 1:5, water bath temperature of 45 °C, and ultrasonic hydration time of 20 min for all subsequent formulations.

#### 3.2.2 Extraction and characterization of NKCM for NK-Lip@Q

While conventional liposomes could improve the water solubility and bioavailability of QUC, they often exhibit limited tumor-targeting capability and a short circulation half-life. To address these limitations, NKCM were employed to endow liposomes with tumor-recognition and immune-evasive properties. NK cells, a type of lymphocyte, could directly lyse tumor cells without prior sensitization, primarily recognizing cells with low expression major histocompatibility complex Class I (MHC I) molecules than normal cells—a common feature of many cancer cells (Abri Aghdam et al., 2019; Gelen et al., 2017). This study compared two extraction methods for NKCM, and the results showed that there was no residual DNA in NKCM1 extracted by method 1, while a small amount of DNA was detected in NKCM2 obtained by method 2 (Fig. 2D). Optical microscope showed that NKCM1 exhibited uniformly size membrane fragments lacking intact cellular morphology (Fig. 2E). Furthermore, DLS measurements showed that NKCM1 averaged 285 nm in size an average with a surface charge of −8.95 ± 0.24 mV (Fig. 2F), indicating that it is a good liposome outer membrane.

#### 3.2.3 Characterization of NK-Lip@Q

**Fig. 3:**
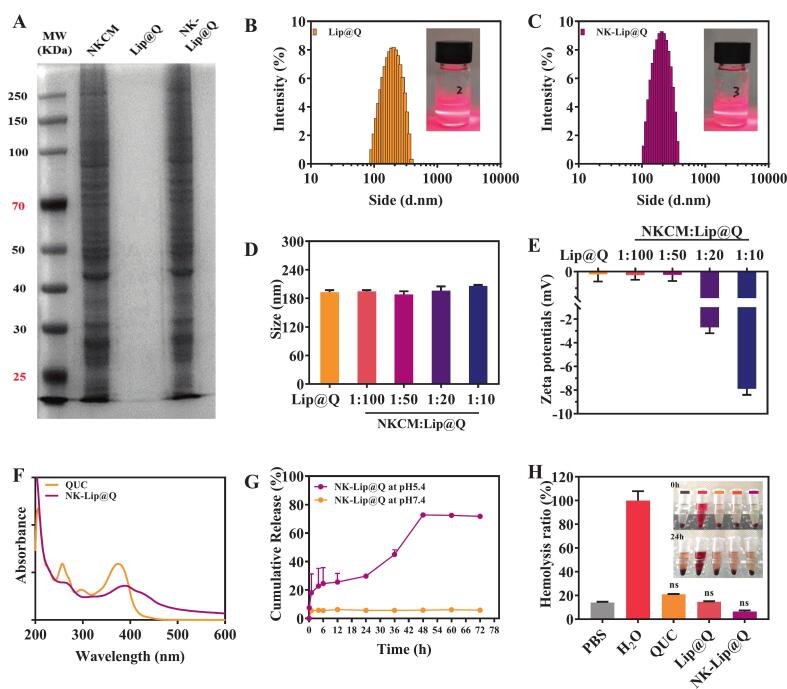
Preparation, characterization, and in vitro evaluation of NK-Lip@Q. (A) SDS-PAGE of NKCM, Lip@Q and NK-Lip@Q. The hydrodynamic size distributions of Lip@Q (B) and NK-Lip@Q (C). The hydrodynamic particle size (D) and Zeta potential (E) of NK-Lip@Q with different ratio of NKCM/Lip@Q. (F) The UV/Vis spectra of QUC and NK-Lip@Q. (G) Cumulative release of quercetin from NK-Lip@Q at two pH conditions. (H) Hemolysis test for QUC, Lip@Q and NK-Lip@Q. (n = 3; ns = no significance, compared to PBS).

NK-Lip@Q was obtained mainly by integrating NKCM into the lipid layer of Lip@Q through the polycarbonate film hydration-extrusion method. The morphological features of NK-Lip@Q were evaluated at both macroscopic and microscopic levels (Fig. 2G-I). NKCM modification did not markedly affect the appearance of Lip@Q solution (Fig. 2G). TEM further confirmed that NKCM modification did not alter the spherical morphology or surface uniformity of Lip@Q (Fig. 2H&I). SDS-PAGE analysis revealed that NK-Lip@Q exhibited protein bands similar to those of NKCM (Fig. 3A). These findings suggested that NKCM were successfully integrated into the structure of liposome. Such structural integration is expected to endow NK-Lip@Q with enhanced biomimetic properties and potential tumor-targeting ability, consistent with previous report on NK cell membrane infused biomimetic liposomes (Pitchaimani et al., 2018).

Given that NKCM-related surface proteins contribute to tumor recognition, while nanoscale size and surface charge are critically influence the colloidal stability and in vivo circulation, NK-Lip@Q with different NKCM/Lip@Q ratios were prepared and systematically characterized (Fig. 3B-E). When the NKCM/Lip@Q ratio increased from 1:50 to 1:10, the average particle diameter of NK-Lip@Q increased by approximately 9 to 20 nm, and the Zeta potential gradually approached that of NKCM (−8.95 ± 0.24 mV). According to Németh et al. (2022), particles with moderate negative surface charge and vesicle sizes around 150 nm tend to exhibit enhanced tumor accumulation. Therefore, NK-Lip@Q with an NKCM/Lip@Q ratio of 1:10 was chosen for subsequent in vitro and in vivo evaluations based on its optimal balance between particle size and surface potential. The particle size of NK-Lip@Q was 206.36 ± 1.81 nm, and its Zeta potential was −7.91 ± 0.50 mV.

To further verify successful drug encapsulation, ultraviolet-visible spectroscopy was performed. Free QUC exhibited characteristic absorption peaks at 257 nm and 375 nm, whereas NK-Lip@Q showed a single broadened peak at 390 nm with attenuated intensity. This spectral shift indicated that QUC was effectively encapsulated within the lipid bilayer, leading to the masking of its intrinsic absorbance bands. The EE% of QUC in NK-Lip@Q reached 60.69 ± 1.32 %, demonstrating satisfactory drug loading capacity and structural integrity of the liposomes.

#### 3.2.4 pH-responsive release behavior and hemocompatibility of NK-Lip@Q

**Fig. 4:**
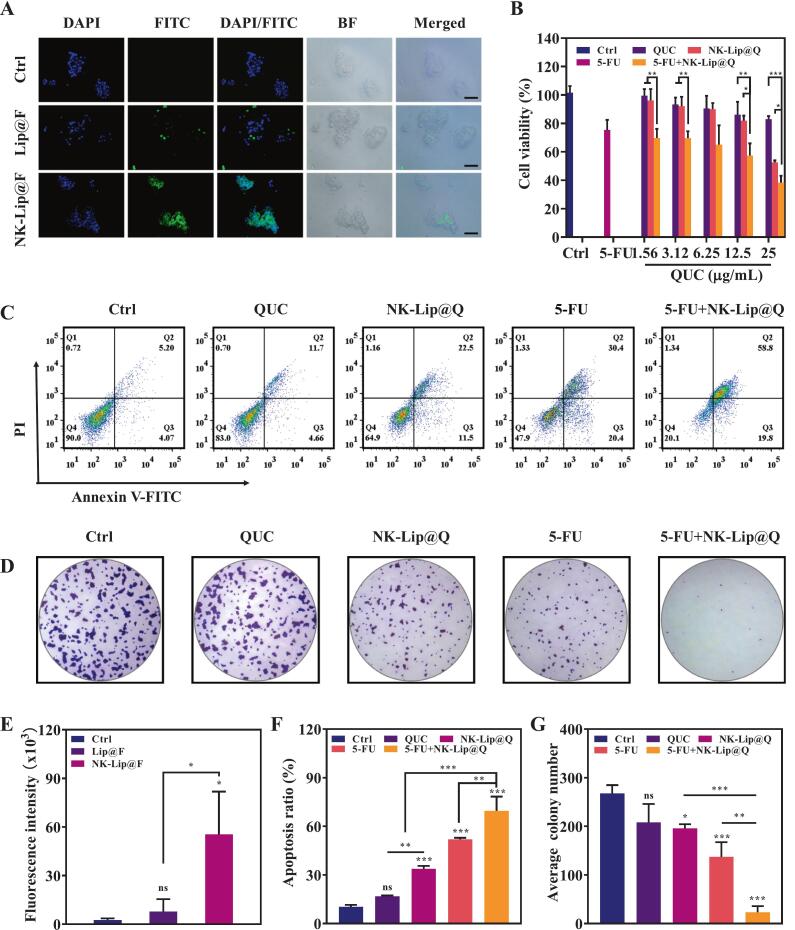
In vitro antitumor effects of NK-Lip@Q against gastric cancer N87 cells. (A) Fluorescence microscopy images of the cellular uptake of Lip@F and NK-Lip@F on N87 cells. (B) Cell viability of N87 cells incubated with QUC, NK-Lip@Q, 5-FU and 5-FU + NK-Lip@Q for 24 h. (C) N87 cells were stained with AnnexinV−FITC and PI for cell apoptosis analysis by the flow cytometry after treatment with QUC, NK-Lip@Q, 5-FU and 5-FU + NK-Lip@Q. (D) Representative colony formation pictures of N87 cells in each group. Quantitative analysis of the relative fluorescence intensity (E), apoptosis ratio (F) and average colony number (G) were also calculated. (Scale bar: 100 μm; *n* = 3; *P < 0.05, **P < 0.01, ***P < 0.001, ns = no significance, compared to Ctrl).

To assess pH-triggered release, NK-Lip@Q was incubated at pH 7.4 (physiological environment) or pH 5.4 (acidic tumor microenvironment) for 72 h and cumulative QUC release was quantified. NK-Lip@Q exhibited a pronounced sustained and pH-dependent release profile, a rapid initial burst phase (approximately 25 % within the first 6 h), followed by a sustained release phase, reaching 72.75 ± 0.69 % at 48 h. Under physiological pH 7.4, NK-Lip@Q demonstrated high stability, with a cumulative release of only about 5.82 % throughout the study period (Fig. 4G). This result indicates excellent stability of NK-Lip@Q in circulation, which is critical for minimizing premature drug leakage and off-target effects. The significant difference in release kinetics between the two pH conditions confirms the successful construction of a pH-responsive liposomes. This desirable feature suggests that NK-Lip@Q remains stable in the bloodstream but rapidly releases its QUC upon exposure to the acidic tumor microenvironment, thereby enhancing targeted delivery and therapeutic efficacy. Similar pH-triggered release phenomena have been reported in other acid-responsive liposomes designed for tumor targeting (Fang et al., 2018), supporting the reliability of the observed mechanism.

To comprehensively evaluate the in vitro release behavior of NK-Lip@Q, the cumulative release data were fitted to zero-order, first-order, Higuchi, and Ritger-Peppas kinetic models to identify the optimal release model and elucidate the underlying release mechanism. The results showed that the release profile of NK-Lip@Q was best described by the zero-order kinetic model (R^2^ = 0.9356), indicating excellent sustained-release properties with stable and controllable release behavior. The Ritger-Peppas model yielded an R^2^ value of 0.8838 and an n value of 0.97, suggesting that QUC release from the liposomes primarily followed a Case-II transport mechanism (Danyuo et al., 2019). The slow and continuous degradation of the liposome membrane likely provided a stable diffusion interface, resulting in a constant release rate.

Hemolysis assay was subsequently performed to confirm the blood compatibility of NK-Lip@Q. (Fig. 3H). No hemolysis was observed in the PBS group (negative control), while the H_2_O group (positive control) caused complete erythrocyte lysis, evidenced by the red supernatant after centrifugation. Quantitative analysis revealed that at a quercetin concentration of 50 μg/mL, the hemolysis rates of the PBS, QUC, Lip@Q, and NK-Lip@Q groups were 14.02 ± 0.59 %, 20.91 ± 0.22 %, 14.52 ± 0.54 % and 6.1 ± 1.05 %, respectively. The hemolysis rate of NK-Lip@Q was below 10 %, which is generally regarded as non-hemolytic (Guo et al., 2021), indicating its good blood compatibility.

### 3.3 Antitumor effect of NK-Lip@Q combined with 5-FU in vitro

Before evaluating the anti-tumor efficacy of NK-Lip@Q in vitro, we examined whether the NKCM modification affected the intracellular uptake of FITC-loaded liposomes. The fluorescence intensity in N87 cells treated with of NK-Lip@F was approximately seven times higher than that in Lip@F group, confirming that NKCM modification significantly enhanced tumor-specific uptake and improved the intracellular delivery efficiency of QUC (Fig. 4A&E). MTT resluts showed that as the concentration of NK-Lip@Q increased, cell viability decreased from 67.71 ± 5.99 % to 38.31 ± 4.87 %. The combination of 5-FU and NK-Lip@Q induced significantly higher cytotoxicity than either agent alone (Fig. 4B). The calculated Chou-Talalay combination index (CI = 0.68) indicated a strong synergistic interaction as previously reported (Gelen et al., 2017; Tavakoli Pirzaman et al., 2023). Flow cytometry results further verified this effect, as the ratio of apoptotic cells in the 5-FU + NK-Lip@Q group reached 69.60 ± 8.71 %, markedly higher than in the single-treatment groups (Fig. 4C&F). As shown in Fig. 4D&G, NK-Lip@Q treatment significantly reduced both the number (23.00 ± 12.77 vs. 267.67 ± 17.01 in control) and size of cell colonies, indicating potent suppression of clonogenic potential. The combined treatment of NK-Lip@Q and 5-FU further suppressed the formation of tumor cell colonies, indicating a pronounced inhibitory effect on the long-term proliferative capacity of N87 cells. NK-Lip@Q and 5-FU exerted synergistic antitumor effects through complementary mechanisms: 5-FU primarily disrupts DNA synthesis in tumor cells (Yuan et al., 2023), while QUC in NK-Lip@Q, as described in Section 3.1, downregulates cell cycle-related genes (CCNB1, CDK1, and CCNA2), thereby interfering with cell cycle progression, promoting apoptosis, and ultimately reducing proliferative activity. The interplay of these distinct mechanisms significantly enhanced the overall tumor growth inhibition. Moreover, similar synergistic effects between QUC and drugs have been reported in other nanotherapeutic co-delivery systems (Xu et al., 2021; Sun et al., 2022), further supporting the compatibility and therapeutic potential of this combined delivery strategy.

### 3.4 Antitumor effect of NK-Lip@Q combined with 5-FU in vivo

#### 3.4.1 Antitumor effect of NK-Lip@Q + 5-FU in orthotopic gastric cancer mouse model

**Fig. 5:**
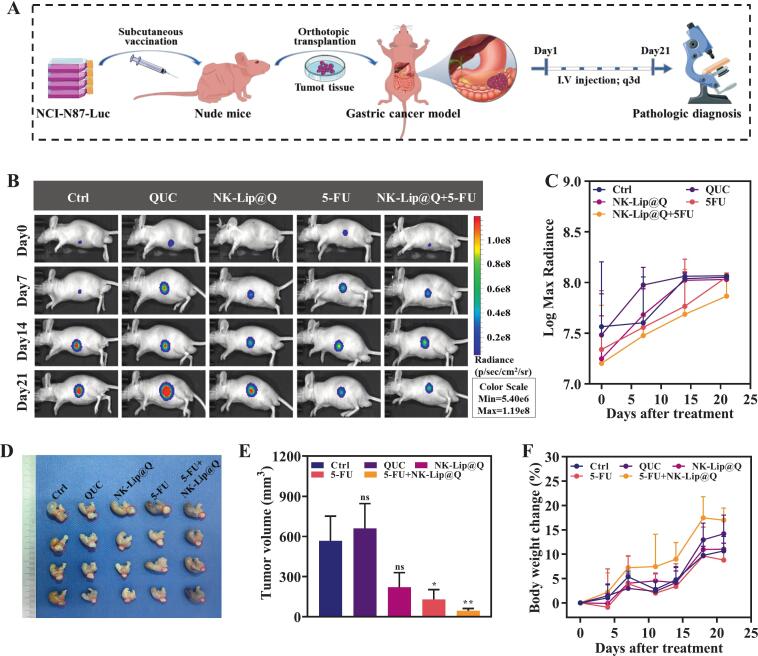
Therapeutic evaluation of NK-Lip@Q and 5-FU combination therapy in vivo. (A) Schematic diagram of the gastric cancer model construction and the 5-FU combined with NK-Lip@Q therapeutic protocol. In vivo imaging images (B) and quantitative analysis (C) of mice with gastric cancer in each treatment cycle. (D) The photograph images of gastric tumors collected from mice at the end of the experiment. E) Gastric tumor volume change in mice subjected to various therapeutic regimens. (F) Body weight alterations in tumor-bearing mice. (*n* = 7; *P < 0.05, **P < 0.01, ***P < 0.001, ns = no significance, compared to Ctrl).

To further evaluate the therapeutic efficacy, an orthotopic gastric cancer model was established using luciferase-labeled NCI-N87-Luc cells, and tumor progression was dynamically monitored by bioluminescence imaging (Fig. 5A). As shown in Fig. 5B&C, bioluminescence signals in all groups were primarily localized in the gastric region, and the signal intensity gradually increased with the extension of treatment. Visually, the control and QUC groups exhibited the strongest bioluminescence signals. However, quantitative analysis based on Log Max Radiance revealed no significant differences in signal intensity among the groups (ns, *P* > 0.05). As shown in Fig. 5D&E, the tumor volume in the control group reached approximately 600 mm^3^ after treatment. Neither 5-FU nor QUC alone significantly inhibited tumor growth compared with the control group (ns, *P* > 0.05). In contrast, treatment with NK-Lip@Q led to marked reduction in tumor volume (*P* < 0.05), indicating its intrinsic antitumor efficacy. Remarkably, the 5-FU + NK-Lip@Q group exhibited the most pronounced inhibitory effect, achieving a tumor inhibition rate of 92.26 % and an average tumor volume of 45.38 ± 16.45 mm^3^ (*P* < 0.01). These results demonstrate a strong synergistic antitumor effect between NK-Lip@Q and 5-FU, suggesting that co-administration effectively enhances chemotherapeutic efficacy.

**Fig. 6:**
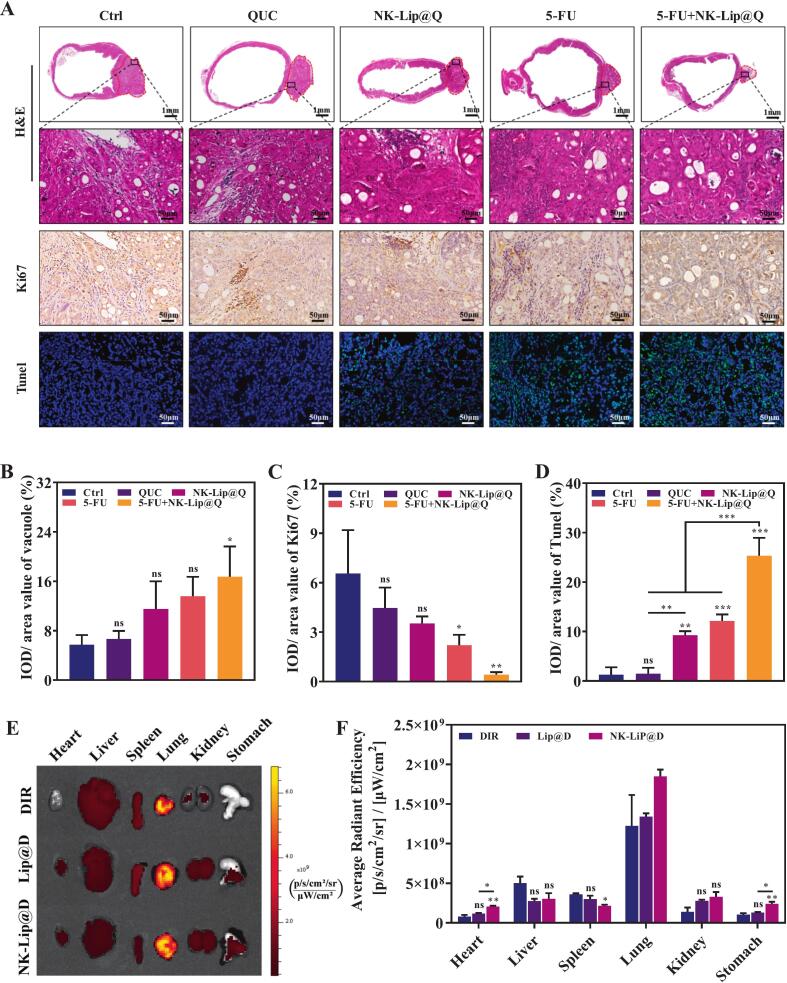
Histological analysis of antitumor effects and in vivo targeting of NK-Lip@Q. (A) Representative micrographs of H&E, Ki67 and Tunel staining in mice exposed to different drug treatments. (B) Nucleus, (C) Ki67, and (D) TUNEL levels were semi-quantified by using Image J (*n* = 4). (E) In vivo imaging of mice after intravenous injection of DIR, Lip@D and NK-Lip@D. (F) The quantitative analysis of relative fluorescence intensity in heart, liver, spleen, lung, kidney, and stomach (*n* = 3). (*P < 0.05, **P < 0.01, ***P < 0.001, ns = no significance, compared to Ctrl).

Histopathological and immunofluorescent analyses provided further evidence of the therapeutic synergy. H&E staining revealed pronounced vacuolization, nucleolysis, and disruption of cellular architecture in the tumor tissues of the 5-FU + NK-Lip@Q group compared with the Ctrl group (Fig. 6 A&B). Moreover, Ki67 immunostaining showed a marked reduction in proliferative activity following combination treatment (Fig. 6A&C), indicating effective inhibition of tumor cell proliferation. TUNEL fluorescence staining showed a gradual increase in apoptotic signals (green fluorescence) from the NK-Lip@Q group to the 5-FU + NK-Lip@Q group (Fig. 6A&D). Among them, the 5-FU + NK-Lip@Q group exhibited the highest levels of apoptosis. These findings suggested that QUC-loaded biomimetic liposomes enhanced 5-FU-induced apoptosis, likely through promoting cell cycle arrest and DNA fragmentation (Zhao et al., 2020). Overall, the combination of NK-Lip@Q and 5-FU achieved superior tumor suppression via synergistic pathways involving both inhibition of DNA synthesis and regulation of the cell cycle.

**Fig. 7:**
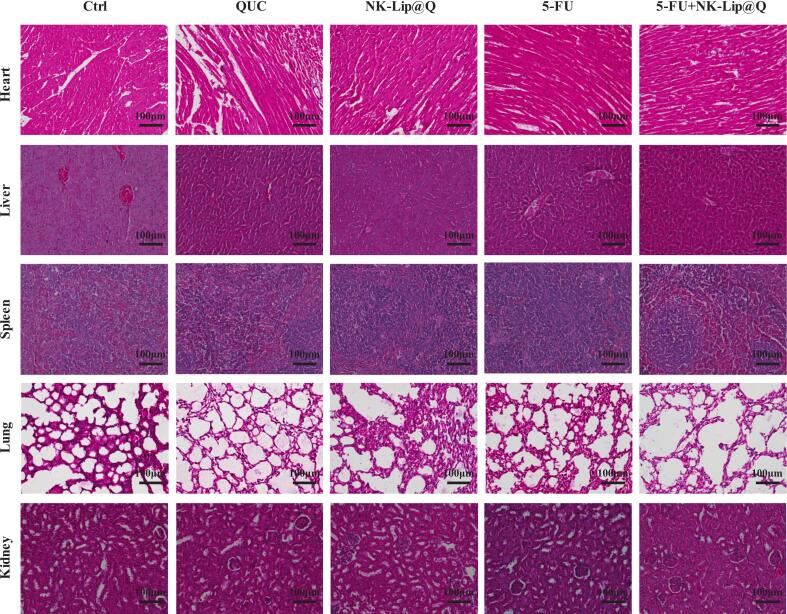
Representative microscopic images of H&E staining of internal organs in mice treated with different drugs.

H&E sections of heart, liver, spleen, lung and kidney showed no treatment-related lesions in any group (Fig. 7). These findings suggest that the combined administration of NK-Lip@Q and 5-FU induces no evident systemic toxicity and well tolerated in vivo.

#### 3.4.2 In vivo biodistribution

To evaluate the tumor-targeting efficiency of NK cell membrane–modified liposomes in gastric cancer, ex vivo fluorescence imaging of major organs was performed. As shown in Fig. 6E&F, the three formulations (free DIR, Lip@D, and NK-Lip@D) displayed distinct biodistribution profiles across the heart, liver, spleen, lungs, kidneys, and orthotopic gastric tumors. Among them, NK-Lip@D exhibited the strongest and most specific accumulation in gastric tumor tissues, with a significantly higher average radiant efficiency than that of free DIR and Lip@D (*P* < 0.05). This result directly demonstrated that NKCM modification effectively enhanced the tumor-targeted delivery of liposomes to gastric cancer sites. In addition, the incorporation of liposomes markedly altered the biodistribution pattern of DIR in vivo. Compared with the free DIR group, both Lip@D and NK-Lip@D showed significantly increased fluorescence signal intensity in the heart (*P* < 0.05), which may be attributed to the prolonged systemic circulation of liposomes, leading to enhanced background signals in the highly perfused cardiac tissue (Ernsting et al., 2013). Interestingly, NK-Lip@D showed a reduced fluorescence signal intensity in the spleen, likely because membrane proteins on the NK cell surface facilitated immune evasion from hepatic and splenic macrophage capture (Pitchaimani et al., 2018). Another notable finding was the higher accumulation of NK-Lip@D in the lungs compared with the other two groups, which may be related to certain protein characteristics on the NK membrane or the retention effect of pulmonary capillaries on nanoparticles (Ozsoy et al., 2024). Taken together, these ex vivo biodistribution data strongly indicate that NK-Lip can efficiently deliver its QUC to gastric tumor tissues, demonstrating excellent tumor-targeting potential.

### 3.5 Limitations

The preparation process of Lip@QUC was relatively simple. However, the extraction, purification, and fusion of NKCM may restrict its feasibility for industrial-scale production, which warrants further optimization using standardized biomanufacturing techniques.

Several additional limitations should also be acknowledged. First, the relatively small number of animals used in the in vivo experiments may limit the statistical power and generalizability of the findings. Second, the in vivo tumor-targeting ability of NK-Lip@QUC was assessed solely through fluorescence imaging. Although this method intuitively demonstrates the liposome targeting capability, quantitative determination of drug concentrations in target tissues remains the gold standard for evaluating biodistribution. Future studies will systematically quantify the organ distribution and further verify the tumor-targeting performance of NK-Lip@QUC. Third, the mechanistic investigation in this study was relatively preliminary, focusing primarily on cellular-level effects. In further work, detailed molecular-level analyses of tumor tissues following NK-Lip@Q treatment and mechanistic validation of NK-Lip-mediated drug delivery will be conducted. Finally, although NK-Lip@QUC showed good biocompatibility in vivo, systematic toxicity studies (including hematological evaluations) and long-term safety evaluations are still required before clinical translation.

## 4 Conclusions

In this study, we successfully developed bioinspired, pH-sensitive liposomes (NK-Lip@Q). Quercetin was precisely delivered to gastric cancer tissues due to the tumor targeting function of NKCM. Once the intratumoral accumulation of quercetin reached an effective threshold, it down-regulated the expression of cycle-related proteins and enhanced the tumor-killing effect of 5-FU through mechanism complementarity. Therefore, the combination of NK-Lip@Q and 5-FU holds promise as a potential strategy for gastric cancer, warranting further investigation.

## List of Chemical Compounds

**Quercetin:** 3,3′,4′,5,7-pentahydroxyflavone, QUC

**5-fluorouracil:** 5-fluoro-1H-pyrimidine-2,4-dione, 5-FU

## CRediT authorship contribution statement

**Qinghua Lan:** Methodology, Funding acquisition, Data curation. **Miao Wang:** Visualization, Methodology, Data curation. **Yanyan Zhu:** Formal analysis, Conceptualization. **Xiayan Zhang:** Methodology, Data curation. **Ruolei Ye:** Methodology, Data curation. **Zhengbo Wu:** Data curation, Writing – review & editing. **HaiCi Lan:** Methodology, Data curation. **Songmei Luo:** Project administration, Methodology, Formal analysis. **Yanyan Xu:** Validation, Supervision, Funding acquisition, Data curation, Writing – review & editing. **qinghua Lan:** Writing – original draft.

## Informed consent statement

Not applicable.

## Disclosure statement

No potential conflict of interest was reported by the authors.

## Funding

This work was supported by the 10.13039/501100004731Natural Science Foundation of Zhejiang Province under grant no.[LBY22H280006] & [LQ23H110001]; the Zhejiang Medical and Health Science and Technology Projects under grant no. [2023RC117] & [2024KY1855]; and the Zhejiang Province Traditional Chinese Medicine Science and Technology Projects under grant no.[2023ZR143].

## Declaration of competing interest

The authors declare that they have no known competing financial interests or personal relationships that could have appeared to influence the work reported in this paper.

## Data Availability

Data will be made available on request.
